# COVID-19 related risk factors and their association with non-syndromic orofacial clefts in five Arab countries: a case-control study

**DOI:** 10.1186/s12903-023-02934-y

**Published:** 2023-04-28

**Authors:** Heba Jafar Sabbagh, Rana A. Alamoudi, Mohammad Zeinalddin, Taimoor Al Bulushi, Ola B. Al-Batayneh, Mamdouh A. AboulHassan, Mohamed Koraitim, Maryam Quritum, Buthaina Almuqbali, Sultan Musaad Alghamdi, Shaimaa Mohsen Refahee, Lateefa Alkharafi, Fatemah Fahad Taqi, Bader Albassam, Mariam Ayed, Alia Embaireeg, Raqiya Alnahdi, Mona Talal AlSharif, Fatma Dawood Abdulhameed, Aziza Johar Aljohar, Najla Sulaiman Alrejaye, Manal Ibrahim Almalik, P S Viswapurna, Tamara Al Halasa, Maha El Tantawi

**Affiliations:** 1 Pediatric Dentistry Department, Faculty of Dentistry, King Abdulaziz University, Jeddah, 21589 Saudi Arabia; 2 Private Practice, Muscat, Oman; 3 Plastic and Craniofacial Surgery, Khoula Hospital, Muscat, Oman; 4 Preventive Dentistry Department, Jordan University of Science & Technology, Irbid, 22110 Jordan; 5 Pediatric Plastic Surgery Department, Cairo University, Cairo, Egypt; 6 Maxillofacial and Plastic Surgery Department, Faculty of Dentistry, Alexandria University, Alexandria, Egypt; 7 Department of Pediatric Dentistry and Dental Public Health, Faculty of Dentistry, Alexandria University, Alexandria, 21527 Egypt; 8 Ministry of Health, Khoula Hospital, Muscat, Oman; 9 Ministry of Health, Bisha, 67711 Saudi Arabia; 10 Oral and Maxilofacial Surgery, Faculty of Dentistry, Fayoum University, Faiyum, Egypt; 11 Cleft and Craniofacial Unit, Ministry of Health, Kuwait, Kuwait; 12 Department of General Dentistry, Ministry of Health, Kuwait, Kuwait; 13 Neonatal Department, Maternity Hospital-Kuwait, Kuwait, Kuwait; 14 Oman Dental College, Muscat, Oman; 15 Department of Dental Public Health, Faculty of Dentistry, King Abdulaziz University, Jeddah, 21589 Saudi Arabia; 16 Pediatric Surgery Department, King Salman Medical City, Maternity and Children’s Hospital, Madinah, Saudi Arabia; 17 Department of Dentistry, King Faisal Specialist Hospital and Research Center, Riyadh, Saudi Arabia; 18 Department of Dentistry, King Abdulaziz Medical City, Ministry of National Guard Health Affairs, College of Dentistry, King Saud bin Abdulaziz University for Health Sciences, King Abdullah International Medical Research Center, Riyadh, Saudi Arabia; 19 Dental Department, King Fahd Armed Forces Hospital, Jeddah, Saudi Arabia

**Keywords:** Non-syndromic orofacial cleft, Etiology, Stress, COVID-19, Fear, Life events, Risk factors

## Abstract

**Background:**

The environmental etiology of non-syndromic orofacial clefts (NSOFCs) is still under research. The aim of this case-control study is to assess COVID-19 associated factors that may be related to the risk of NSOFC in five Arab countries. These factors include COVID-19 infection, COVID-19 symptoms, family member or friends infected with COVID-19, stress, smoking, socioeconomic status and fear of COVID-19.

**Methods:**

The study took place in governmental hospitals in five Arab countries from November 2020 to November 2021. Controls are matched in the month of delivery and site of recruitment. A clinical examination was carried out using LASHAL classification. Maternal exposure to medication, illnesses, supplementation, COVID-19 infection during their pregestation and 1st trimester periods were evaluated using a validated questionnaire. Maternal exposure to stress was assessed using the Life Events scale, fear of covid-19 scale, family member or friend affected with covid-19 infection, pregnancy planning and threatened abortion.

**Results:**

The study recruited 1135 infants (386 NSOFC and 749 controls). Living in urban areas, maternal exposure to medications 3-months pregestation, maternal exposure to any of the prenatal life events and maternal fear of COVID-19 significantly increased the risk of having a child with NSOFC. On the other hand, mothers exposed to supplementation 3-months pregestation, mothers suspected of having COVID-19 infection, family members or friends testing positive with COVID-19 significantly decreased the risk of having a child with NSOFC.

**Conclusions:**

This study suggests that NSOFC may be associated with maternal exposure to lifetime stress and COVID-19 fear in particular, with no direct effect of the COVID-19 infection itself. This highlights the importance of providing psychological support for expecting mothers during stressful events that affect populations such as the COVID-19 pandemic, in addition to the usual antenatal care.

**Supplementary Information:**

The online version contains supplementary material available at 10.1186/s12903-023-02934-y.

## Introduction

Non-syndromic orofacial clefts (NSOFCs) including cleft to the lips and/or palate, can lead to several functional and aesthetic problems including difficulties in feeding especially at birth, speech and hearing difficulties, and swallowing and nasal regurgitation problems [1]. Cleft defects can be surgically repaired in early childhood. Unrepaired clefts cause deformity due to abnormal facial development and scarring with long-lasting functional and psychosocial problems [2]. Affected children have a higher morbidity and mortality rate throughout their lives than unaffected counterparts. Thus, clefts have a long-lasting adverse impact on the health and social well-being of affected individuals [3–5].

Many factors contribute to NSOFC including genetics [6–9], which plays a main role in the development of NSOFC, environmental, and stress-environmental interaction risk factors [10]. The interaction of these factors and their relation with NSOFC etiology is complex. Epidemiological and experimental evidence suggests that environmental risk factors are important in the etiology of NSOFC, including viral infection, poor nutrition, exposure to tobacco smoke, alcohol, medications, and stress in early pregnancy [2]. Prenatal maternal stress during pregnancy has been found to affect the fetal environment and was linked to poor birth outcomes including low birth weight, preterm birth, infant mortality, and birth defects such as NSOFCs [11, 12]. The mechanism behind this is not yet clear. However, it could be explained by the rise of blood cortisol levels or inflammatory activity [13, 14] .

The COVID-19, caused by the severe acute respiratory syndrome coronavirus 2, is the most serious global health threat of the 21st century [15]. According to the World Health Organization (WHO), COVID-19 has killed almost three million people across the world up to early 2021 [15, 16] with severe economic, social, environmental, and health challenges. The highly infectious virus spreads from person to person through close contact and droplets causing mild, non-specific, or severe symptoms [17] ranging from cough, cold, fever, tiredness, and bone pain, to shortness of breath and severe pneumonia with respiratory failure and septic shock [18, 19]. SARS-CoV-2 was found to be expressed in saliva [20, 21]. Additionally, SARS-CoV-2 has been suggested to result in congenital anomalies by invading human cells’ Angiotensin-converting enzyme receptor 2 (ACE2). ACE2 receptors are found in the gametes, zygotes, uterus, and developing fetus and may result in neurodevelopmental malformations [22]. However, in their international observational cohort study, Hernandez et al. found no teratogenic effects of maternal SARS-CoV-2 infection [23]. Nevertheless, the teratogenic potential effect of the virus still warrants further studies [23].

COVID-19 has had a tremendous psychological effect, owing to fear of its unknown nature and worrying for one’s and loved ones’ health [24–26]. There is also a link between medical history, depression and anxiety due to the spread of COVID-19 [15, 19]. COVID-19 may also be associated with lifestyle changes such as smoking [27, 28], which is an important risk factor for NSOFCs [29] and some subgroups, such as healthcare professionals may be at higher risk of catching the COVID-19 infection [30, 31]. The interaction of these factors may affect the risk of NSOFCs and it is important to assess these relations.

The aim of this multi-country case-control study is to assess COVID-19 associated factors that may be related to the risk of NSOFC in five Arab countries. These factors include COVID-19 infection status and fear, lifetime events, smoking, and exposure to medication, illness, supplementation and smoking. The hypothesis of the study was that COVID-19 infection and fear would be associated with NSOFC even when other risk factors are considered.

## Methods

### Design and ethical considerations

Institutional Review Board approval was obtained from King Abdulaziz University (257-07-21), the Ministry of Health (H-02 J-022), King Fahad Medical City (20–642), King Abdullah International Medical City (H-01-R-005), in Saudi Arabia, as well as Jordan University of Science and Technology (104/147/2022) in Jordan. A formal consent with written information about the study purpose, methods and data confidentiality was signed by parents of all participating children in all study sites.

A case-control study design was used to compare cases (infants with NSOFCs) to controls (unaffected newborns) recruited from December 2020 to December 2021. The date was selected 9 months after the date of the Saudi Ministry of Health COVID-19 lockdown (March-2020) [32].

The study took place in governmental hospitals (maternity or cleft referral centers) in five Arab countries: Saudi Arabia (27-hospitals distributed in Saudi Arabia’s five regions), Egypt (two-referral centers in Cairo and Alexandria), Oman (The-main cleft referral center in Oman: Khaula Hospital), Kuwait (The two-main governmental referral cleft clinics: Orthodontic cleft and craniofacial orthodontic clinics) and Jordan (main referral cleft clinics for Amman and Irbid).

### Participants

This study included infants born to mothers exposed to the COVID-19 pandemic during their pregestation and/or 1st trimester periods. Further inclusion criteria included cases affected with NSOFC and healthy controls matched to cases by age and location of recruitment. The controls were recruited at the same period of cases; from the vaccination clinics or delivery units, from the same hospitals. Children with syndromic orofacial clefts or controls with any medical condition were excluded from the study based on the geneticist’s and pediatrician’s diagnosis.

Sample size was estimated based on 5% alpha error and 80% study power to detect the difference between cases and controls in exposure to a major risk factor such as stress = 39.58% and 21.13% [33] with sampling ratio = 1:2. The required number of participants was 87 cases and 174 controls. We included all available cases with NSOFCs per site during the data collection period to maximize power and make up for incomplete records or non-response.

### Data collection

When an infant was born with NSOFCs or visited the NSOFC referral clinics, in the study sites during the study period, the site investigator was contacted and the child and parents were scheduled for an appointment with the site investigator in the same week during the infant’s hospital stay, to fill out the paper-based questionnaire. Confirmation of the diagnosis was done to exclude syndromic cases. Selection of age -matched controls was done by the site investigator at the same time, from the same hospital. Data was collected using a clinical examination and a questionnaire.

### Clinical examination

A clinical examination was carried out using mirror and illumination light in the neonatal intensive care unit where infants with NSOFCs were admitted, or in the plastic surgery clinics. The LASHAL; i.e. Lip, Alveolus, Soft Palate, Hard Palate, Alveolus and Lip; classification system [34] was used to categorize the infants. NSOFCs cases were grouped to cleft lip with or without palate (CL/P) and Cleft palate alone (CP).

### Questionnaire

Parents of cases and controls were interviewed using a tool based on the WHO questionnaire for congenital anomalies. It was validated in a previous cleft study in a Saudi population [35] in Arabic. The questionnaire included several sections. In Sect. 1, socio-demographic information was collected including country of residence, type of residence area (including three categories: (a) metropolis/industry if population > one-million or industrial within 10-kilometer from industries or factories, (b) urban with population of at least 500,000, and (c) rural with population < 100,000), child sex (male or female), parents’ highest educational level (classified into less than high school (none or primary school), high school and greater than high school (university or higher)), family monthly income (classified into low if “in debt”; moderate if “just meet routine expenses” and high if “meet routine expenses and emergencies or able to save”.

The second section assessed maternal exposure to risk factors during pregestation and the pregnancy 1st trimester including supplementation, medication and illness, in addition to nicotine exposure, consanguinity and family history of birth defects. There were also questions assessing maternal stress using the validated “Prenatal modified Life Events Scale” [36] which assesses seven stress events that may lead to severe consequences and strain on the mother’s psychological health, such as family pressure, marital status changes, changes in residency, work status changes, family problems, problems with friends and neighbors and robbery. Furthermore, two pregnancy characteristics were included that are suggested to affect a mother’s psychological health during pregnancy. These were, whether the pregnancy was planned and whether there was a threatened abortion [37, 38].

The third section assessed COVID-19 related factors including COVID-19 infection confirmed through a PCR test, suspected COVID-19 infection without testing and if a family member or a friend has been infected with COVID-19 [39] adapted from a questionnaire in Arabic [25]. This section also assessed fear of covid-19 using the Fear of Covid-19 Scale which has been previously validated in English and Arabic. The questionnaire includes seven yes/no questions with a total score ranging from 7 (no fear) to 14 (maximum fear) [40].

The questionnaire was further validated in this study by 20 parents (face validity) and six experts (content validity). The content validity index was 0.9 and the Cronbach alpha of internal consistency for the Fear of Covid-19 Scale was 0.85.

### Statistical analysis

Data was entered in SPSS Statistics-19 for analysis. Qualitative and quantitative variables were described by frequencies with percentages and means with standard deviations respectively. The groups were compared using the Chi-square test, or Mann-Whitney U test. Regression analysis was used to assess the association of the independent, risk indicators (maternal exposures to medication, illnesses and nicotine, COVID-19 infection and fear, pregnancy factors and maternal exposure to stress life event) to NSOFC (dependent factor) controlling for confounders (sociodemographic factors). Statistical significance was set at 0.05.

## Results

The study recruited 1135 (386 NSOFC and 749 controls) infants from the five Arab countries: 191 NSOFC and 557 controls from Saudi Arabia, 95 cases and 263 controls from Egypt, 47 NSOFC and 100 controls from Oman, 40 NSOFC and 127 controls from Jordan and 13 NSOFC and 41 controls from Kuwait. The ratio of cases to controls was 1 to 1.94.

Table 1 shows the distribution of participants according to socio-demographic factors. The prevalence of males was significantly higher among children with NSOFC than cases (52.8% and 50.5%, p = 0.018). The percentage of children with NSOFC from urban areas was significantly higher than controls (26.4% and 11.6%, P < 0.001). Also, a significantly greater percentage of fathers of children with NSOFC than controls, had less than high school education (18.4% and 13.8%, P = 0.035) and low monthly income (54.2% and 45.0%, P = 0.002).

**Table 1:** Distribution of the participants according to sociodemographic factors (NSOFC = 386 and controls = 749)

		NSOFCs (%)	Controls (%)	P value
Country	Saudi Arabia	191 (49.5)	366 (48.9)	0.867
	Oman	47 (12.2)	100 (13.4)	
	Kuwait	13 (3.4)	28 (3.7)	
	Egypt	95 (24.6)	168 (22.4)	
	Jordan	40 (10.4)	87 (11.6)	
Child sex	Male	204 (52.8)	378 (50.5)	0.018^*^
	Female	182 (47.2)	371 (49.5)	
Maternal education	< high school	72 (18.7)	115 (15.4)	0.637
	high school	183 (47.4)	360 (48.1)	
	> high school	131 (33.9)	274 (36.6)	
Paternal education	< high school	71 (18.4)	103 (13.8)	0.035*
	high school	202 (52.3)	380 (50.7)	
	> high school	113 (29.3)	266 (35.5)	
Place of residence	Metropolis > million or Industrial	214 (55.4)	452 (60.3)	< 0.001^*^
	Urban (about 500,000)	102 (26.4)	87 (11.6)	
	Rural (< 100,000)	70 (18.1)	210 (28.0)	
Monthly income	Low	215 (54.2)	337 (45.0)	0.002*
	Moderate	144 (37.3)	362 (48.3)	
	High	27 (7.0)	50 (6.7)	

*The Chi-square statistic is significant at the 0.05 level

Table 2 shows that there was a significantly higher percentage of family history of birth defects and consanguinity in children with NSOFC than controls (P < 0.001 and P = 0.005 respectively). Also, a significantly higher percentage of medication use at three months pregestation, father’s smoking 3-months pregestation and mother’s smoking in the 1st trimester was reported in the NSOFC group than the controls (P < 0.001, 0.026 and 0.039 respectively). In addition, a higher percentage of pregnancy cases in the group of children with NSOFC than controls were not planned (68.9% and 48.7%, P < 0.001) and had no threatening abortion (96.4% and 92.7&, P = 0.002).

**Table 2:** Maternal exposure to medication, supplementation and illnesses during the pregestation and 1st trimester period

Variables		NSOFC (%)	Control (%)	P value
Maternal exposure to medication, supplementation and illnesses				
Suplementation 3-months pregestation	Yes	68 (17.6)	124 (16.6)	0.651
	No	318 (82.4)	625 (83.4)	
Supplementations 1st trimester	Yes	313 (81.1)	616 (82.2)	0.632
	No	73 (18.9)	133 (17.8)	
Medications 3-months pregestation	Yes	90 (23.3)	113 (15.1)	< 0.001
	No	296 (76.7)	636 (84.9)	
Medications 1st trimester	Yes	41 (10.6)	72 (9.6)	0.591
	No	345 (89.4)	677 (90.4)	
Illness 3-months pregestation	Yes	96 (24.9)	166 (22.2)	0.304
	No	290 (75.1)	583 (77.8)	
Illness 1st trimester	Yes	95 (24.9)	163 (21.8)	0.230
	No	286 (75.1)	586 (78.2)	
Parental nicotine exposure				
Mother smoking during pregestation	Yes	13 (3.4)	11 (1.5)	0.072
	No	373 (96.6)	738 (98.5)	
Father smoking during pregestation	Yes	167 (43.3)	273 (36.4)	0.026^*^
	No	219 (56.7)	476 (63.6)	
Mother smoking during 1st trimester	Yes	8 (2.1)	5 (0.7)	0.039^*,b^
	No	378 (97.9)	744 (99.3)	
Father smoking 1st trimester	Yes	148 (38.3)	253 (33.8)	0.128
	No	238 (61.7)	496 (66.2)	
Family factor				
Family history of birth defects	Yes	132 (34.2)	35 (4.7)	< 0.001^*^
	No	254 (65.8)	714 (95.3)	
Consanguinity	Yes	173 (44.8)	270 (36.0)	0.005^*^
	No	213 (55.2)	479 (64.0)	

*The Chi-square statistic is significant at the 0.05 level

^b^ Fissure exact test for cells with frequency 5 or less

Table 3 shows maternal exposure to COVID-19 factors and stresses in relation to NSOFC. Maternal exposure to COVID-19 infection was not significantly related to NSOFC (P = 0.578). A significantly lower percentage of children in the NSOFC than controls had mothers with suspected COVID-19 infection (11.7% and 28.2%, P < 0.001) and a family member/friend infected with COVID-19 (20.7% and 37.7%, P < 0.001). Also, mothers of children with NSOFC had a significantly higher fear of COVID-19 score than controls (mean (SD) = 9.2 (2.4) and 8.6 (2.1), p < 0.001) and a significant percentage of mothers of children with NSOFC than controls was exposed to a life event (37% and 21.6%, P < 0.001).

**Table 3:** Maternal exposure to COVID-19 factors including infection, life events during COVID-19 pandemic, and fear of COVID-19 participants

Variables			NSOFC (%)		Control (%)	P value	
COVID-19 infection							
Did the mother tested postive with COVID-19	Yes		50 (13.0)		106 (14.2)	0.578	
	No		336 (87.0)		643 (85.8)		
Did the mother suspect she was infected with COVID-19?	Yes		45 (11.7)		211 (28.2)	< 0.001^*^	
	No		341 (88.3)		536 (71.8)		
Did anyone in the family tested positive with COVID-19?	Yes		80 (20.7)		282 (37.7)	< 0.001^*^	
	No		306 (79.3)		467 (62.3)		
Pregnancy characteristic and stress during COVID-19 pandemic							
Was the pregnancy planned?	Yes		120 (31.1)		384 (51.3)	< 0.001*	
	No		266 (68.9)		365 (48.7)		
Threatened abortion	Yes		14 (3.6)		64 (8.5)	0.002*	
	No		372 (96.4)		685 (92.7)		
If yes to any of the prenatal life event 7 items	Yes		143 (37.0)		162 (21.6)	< 0.001^*^	
	No		243 (63.0)		587 (78.4)		
Severity of exposure to life events	Non		243 (63.0)		587 (78.4)	< 0.001*	
	1-item		89 (23.1)		96 (12.8)		
	> 1-item		54 (14)		66 (8.8)		
Total fear of COVID score	Mean ± SD	9.16 ± 2.393		8.56 ± 2.077		< 0.001^*^	

Details of maternal exposures are displayed in supplementary Tables 1 to 3.

Table 4 shows the regression model for the factors associated with NSOFC. Testing positive for COVID-19 infection was not significantly associated with the presence of NSOFC (AOR = 1.225, 95%CI: 0.749–2.005, although being suspected of infection (AOR = 0.479, 95%CI: 0.301–0.765) and having a family member/ friend infected with COVID-19 (AOR = 0.502, 95%CI: 0.339–0.743) were significantly associated with lower odds of NSOFC. Also, fear of COVID-19 was significantly associated with higher odds of NSOFC (AOR = 1.104, 95%CI: 1.031–1.183).

**Table 4:** Regression analysis for the association between NSOFC phenotypes (dependent factor) and socio-demographic variable and COVID-19 related factors (independent factors)

Variable		AOR (95% CI) P value
Gender of the child	Male	1.056 (0.784–1.422) 0.722
	Female	1.000
Monthly income	Low	1.644 (0.890–3.036) 0.112
	Moderate	0.944 (0.515–1.729) 0.851
	High	1.000
Description of thge place they lived	Metropolis	1.061 (0.707–1.594) 0.774
	Urban	2.269 (1.406–3.662) < 0.001^*^
	Rural	1.000
Gestation	< 33 weeks	0.800 (0.320-2.000) 0.633
	33–36 weeks	1.566 (1.035–2.371) 0.034*
	37–42 weeks	1.000
Paternal education	< high school	1.322 (0.682–2.561) 0.408
	high school	1.237 (0.870–1.758) 0.236
	> high school	1.000
Maternal education	< high school	0.982 (0.527–1.829) 0.954
	high school	0.771 (0.533–1.116) 0.168
	> high school	1.000
Suplementation 3-months pregestation	Yes	0.309 (0.144–0.664) 0.003*
	No	1.000
Supplementations 1st trimester	Yes	0.832 (0.555–1.248) 0.373
	No	1.000
Medications 3-months pregestation	Yes	3.550 (1.720–7.326) < 0.001^*^
	No	1.000
Medications 1st trimester	Yes	1.585 (0.855–2.938) 0.144
	No	1.000
Illness 3-months pregestation	Yes	0.955 (0.631–1.444) 0.826
	No	1.000
Illness 1st trimester	Yes	1.140 (0.752–1.728) 0.537
	No	1.000
Did the mother tested positive with COVID-19	Yes	1.225 (0.749–2.005) 0.419
	No	1.000
Did the mother suspect she was infected with COVID-19	Yes	0.479 (0.301–0.765) 0.002*
	No	1.000
Did anyone in the family test positive with COVID-19	Yes	0.502 (0.339–0.743) < 0.001^*^
	No	1.000
Threatened abortion	Yes	0.403 (0.202–0.805) 0.010*
	No	1.000
Was the pregnancy planned?	Yes	0.619 (0.447–0.858) 0.004*
	No	1.000
If yes to any of the prenatal event scale	Yes	2.225 (1.576–3.141) < 0.001^*^
	No	1.000
Relative with birth defects	Yes	11.469 (7.300-18.019) < 0.001^*^
	No	1.000
Consanguinity	Yes	1.109 (0.802–1.534) 0.532
	No	1.000
Mother smoking pregestation	Yes	0.500 (0.138–1.818) 0.293
	No	1.000
Mother smoking during1st trimester	Yes	8.007 (1.419–45.179) 0.018*
	No	1.000
Father smoking pregestation	Yes	1.239 (0.638–2.409) 0.527
	No	1.000
Father smoking during1st trimester	Yes	0.765 (0.388–1.510) 0.440
	No	1.000
Mean of total fear of COVID scale**		1.104 (1.031–1.183) 0.005*

*Significant at P = 0.05

**from 7 (no fear) to 14 (more fear)

Some known risk indicators were significantly associated with higher odds of NSOFC including maternal exposure to medications 3-months pregestation (AOR = 3.550; P < 0.001), smoking during the first trimester (AOR = 8.007, P = 0.018), maternal exposure to any prenatal life events (AOR = 2.225; P < 0.001) in addition to family history of birth defects (AOR = 11.469, P < 0.001). Other factors were associated with lower odds of NSOFC including supplementation 3-months pregestation (AOR = 0.309; P = 0.003), planned pregnancy (AOR = 0.619, P = 0.004) and threatened abortion (AOR = 0.403, P = 0.010).

## Discussion

The study showed that confirmed COVID-19 infection of mothers was not significantly associated with NSOFC in newborns, although suspected infection and infection in family members or friends were associated with lower odds of NSOFC, and fear of COVID-19 was associated with significant but weakly higher odds of NSOFC. Previously suspected risk indicators were also confirmed, including medication and smoking during pregnancy and pre-pregnancy exposure to stresses. The findings, thus, partly support the study hypothesis.

The study sheds light on the impact of COVID-19 on an orofacial anomaly with complex etiology. Several important points are raised. First, the study presents evidence suggesting an impact of COVID-19 on NSOFC due to COVID-19 related fear. This relation was weak, but statistically significant. It persisted after accounting for lifetime exposure to stressful events and stressful events related to current pregnancy at onset, such as whether it was planned and during its course, such as threatened abortion. It also persisted after accounting for COVID-19 infection. Our study, thus, adds to the growing literature documenting the impact of COVID-19 related stresses and fear on health [25, 41, 42]. Previous research showed an association between COVID-related stresses and stress-related oral conditions such as plaque accumulation and gingival inflammation [43] and oral ulcers [44]. These conditions were acquired after birth and reversible with no permanent impact. The present finding suggests an impact on oral and general health that is irreversible, costly to address and manage, indicating a larger effect on health and wellbeing.

Second, the findings show negative significant associations between NSOFC and suspected COVID-19 infection and COVID-19 infection in family members and friends. This may be a reaction to a surrounding or unconfirmed COVID-19 threat leading pregnant mothers to exert additional caution by avoiding risk indicators for NSOFC during the pandemic.

Third, the findings confirm that exposure to stressors over a lifetime is associated with the risk of NSOFC similar to previous studies [45, 46]. The relation between NSOFC and stress was demonstrated in animal studies [47] and in humans using validated questionnaires measuring stress [48]. A systematic review showed an association between NSOFC and personal life events and population level crises such as wars, earthquakes and hurricanes [48], reporting weak positive association. The impact of stress may also explain the association observed in the present study with lower odds of NSOFC in case of planned pregnancy. Unplanned pregnancy may be associated with depression in pregnant mothers [37] that may build stresses leading to NSOFC.

Fourth, the study showed a protective effect of threatened abortion against NSOFC. Previous studies conducted in Iran and Saudi Arabia showed no significant association between pregnancy complication and NSOFC [35, 49]. However, studies from other countries reported an association between threatened abortion and NSOFC [50, 51]. The difference between studies may be attributed to the amount and type of social support offered during the pandemic to pregnant women with threatened abortion [52]. In Arabic countries, such as those included in the present study, the care for pregnant woman is mainly provided by family members [53] because of the larger family size and stronger family relationships [54], as opposed to relying on medical care which was disrupted during the lockdown in the pandemic.

Fifth, this study supports what was previously reported on the relationship between maternal medication, or nicotine use and the risk of having a child with NSOFC [35, 55]. It also supports the prevention ability of maternal supplementation to decrease the risk of NSOFC [56].

In this study we included five counties with similar ethnicity backgrounds [57] during a pandemic that affected human life dramatically [28, 57, 58] to evaluate the association between stress and NSOFC.

This study has some limitations such as recall bias. In order to decrease the effect of this bias, we recruited the sample as soon as the infant was born, to decrease the duration between the exposures and the time of data recruitment. Another limitation is that the study was conducted during the pandemic which caused some difficulties and delayed data recruitment in some hospitals in two of the included countries. In addition, some questions assessing family problems and lifestyle such as smoking, address issues that are not approved by society and that may likely be under-reported [59]. However, this type of bias is expected from both cases and controls [60, 61], and is thus, unlikely to affect the conclusions. We recommend a prospective cohort study that follows up on mothers during their pregnancy to better support causality. In addition, studies in other part of the world is still warranted as the etiology and prevalence of NSOFC varies globally [62]. Nevertheless, this study evaluates multiple maternal stressors and was, in addition, designed to capture the impact of a population-level disaster in the Arabic counties.

## Conclusion

This study suggests that NSOFC may be associated with maternal exposure to lifetime stress and to COVID-19 fear in particular, with no direct effect of the COVID-19 infection itself. This highlights the importance of providing psychological support for expecting mothers during stressful events that affect populations such as the COVID-19 pandemic, in addition to the usual antenatal care.

## Electronic supplementary material

Below is the link to the electronic supplementary material.

Supplementary Material 1

## Data Availability

All data generated or analysed during this study are included in this published article and its supplementary information files.

## List of Abbreviations

NSOFCs: Non-syndromic orofacial clefts
CL/P: Cleft lip with or without palate
CP: Cleft palate alone
COVID-19: Corona virus disease 2019
LASHAL: Lip, Alveolus, Soft Palate, Hard Palate, Alveolus, and Lip; classification system
