# Supplementary material for: COVID-19 related risk factors and their association with non-syndromic orofacial clefts in five Arab countries: a case-control study
Source: BMC Oral Health. 2023 Apr 28;23:246. doi: 10.1186/s12903-023-02934-y (PMC10141804; doi:10.1186/s12903-023-02934-y)
Supplement: Supplementary file 1 — Supplementary Material 1 [file 12903_2023_2934_MOESM1_ESM.docx]

**Supplementary table 1.** Distribution of the sample according to maternal exposure supplementation.

| **Variables** | | **NSOFC (%)** | **Controls (%)** | **P value** |
| --- | --- | --- | --- | --- |
| Folic acid 3-months pregestation | Yes | 62 (16.1) | 99 (13.2) | 0.193 |
|  | No | 324 (83.9) | 650 (86.8) |  |
| Multivitamins 3-months pregestation | Yes | 36 (9.3) | 62 (8.3) | 0.551 |
|  | No | 350 (90.7) | 687 (91.7) |  |
| Iron 3-months pregestation | Yes | 33 (8.5) | 67 (8.9) | 0.447 |
|  | No | 352 (91.2) | 682 (91.1) |  |
| Folic acid in the 1^st^ trimester | Yes | 299 (77.5) | 682 (91.1) | 0.573 |
|  | No | 87 (22.5) | 180 (24.0) |  |
| Multivitamins in the 1^st^ trimester | Yes | 202 (52.3) | 304 (40.6) | <0.001^*^ |
|  | No | 184 (47.7) | 445 (59.4) |  |
| Iron in the 1^st^ trimester | Yes | 222 (57.5) | 367 (49.0) | 0.007^*^ |
|  | No | 164 (42.5) | 382 (51.0) |  |

**Supplementary table 2 .** Distribution of the sample according to type maternal illnesses.

| **Variables** | | **NSOFC (%)** | **Controls (%)** | **P value** |
| --- | --- | --- | --- | --- |
| Flu pregestation | Yes | 38 (9.8) | 62 (8.3) | 0.378 |
|  | No | 348 (90.2) | 687 (91.7) |  |
| Fever pregestation | Yes | 26 (6.7) | 29 (3.9) | 0.033^*^ |
|  | No | 360 (93.3) | 720 (96.1) |  |
| HBP pregestation | Yes | 10 (2.6) | 10 (1.3) | 0.128 |
|  | No | 376 (97.4) | 739 (98.7) |  |
| Diabetes pregestation | Yes | 12 (3.1) | 31 (4.1) | 0.389 |
|  | No | 374 (96.9) | 718 (95.9) |  |
| Convulsion pregestation | Yes | 17 (4.4) | 32 (4.3) | 0.918 |
|  | No | 369 (95.6) | 717 (95.7) |  |
| Depression pregestation | Yes | 15 (3.9) | 15 (2.0) | 0.061 |
|  | No | 371 (96.1) | 734 (98.0) |  |
| Asthma pregestation | Yes | 4 (1.0) | 14 (1.9) | 0.328^b^ |
|  | No | 382 (99.0) | 735 (98.1) |  |
| Flu 1^st^ trimester | Yes | 40 (10.4) | 43 (5.7) | <0.001^*^ |
|  | No | 341 (88.3) | 706 (94.3) |  |
|  | 99 | 5 (1.3) | 0 (0.0) |  |
| Fever 1^st^ trimester | Yes | 49 (12.7) | 35 (4.7) | <0.001^*^ |
|  | No | 337 (87.3) | 714 (95.3) |  |
| HBP 1^st^ trimester | Yes | 7 (1.8) | 15 (2.0) | 0.827 |
|  | No | 379 (98.1) | 734 (98.0) |  |
| Diabetes 1^st^ trimester | Yes | 11 (2.8) | 57 (7.6) | 0.001^*^ |
|  | No | 375 (97.2) | 692 (92.4) |  |
| Depression 1^st^ trimester | Yes | 16 (4.1) | 16 (2.1) | 0.053 |
|  | No | 370 (95.9) | 733 (97.9) |  |
| Convulsion 1^st^ trimester | Yes | 25 (6.5) | 29 (3.9) | 0.141 |
|  | No | 361 (93.5) | 720 (96.1) |  |
| Asthma 1^st^ trimester | Yes | 7 (1.8) | 1 (0.1) | 0.002^*^ |
|  | No | 379 (98.1) | 748 (99.9) |  |

**Supplementary table 3.** Distribution of the sample according to maternal exposure to stress events and fear.

| Variables | | | NSOFC (%) | Controls (%) | **P value** |
| --- | --- | --- | --- | --- | --- |
| Was the family under pressure | Yes | | 83 (21.5) | 119 (15.9) | 0.019^*^ |
|  | No | | 303 (78.5) | 630 (84.1) |  |
| Marital status change | Yes | | 18 (4.7) | 24 (3.2) | 0.345 |
|  | No | | 368 (95.3) | 725 (96.8) |  |
| Change in family residency | Yes | | 37 (9.6) | 38 (5.1) | 0.004^*^ |
|  | No | | 349 (90.4) | 711 (94.9) |  |
| Work state leave or change | Yes | | 35 (9.1) | 325 (4.3) | 0.001^*^ |
|  | No | | 351 (90.9) | 717 (95.7) |  |
| Problems with the family inside home | Yes | | 44 (11.4) | 44 (5.9) | 0.001^*^ |
|  | No | | 342 (88.6) | 705 (94.1) |  |
| Problems with friends and neighbors | Yes | | 6 (1.6) | 14 (1.9) | 0.7503 |
|  | No | | 380 (98.4) | 735 (98.1) |  |
| Was the family robbed? | Yes | | 7 (1.8) | 0 (0.0) | 0.001^*^ |
|  | No | | 379 (98.2) | 749 (100.0) |  |
| Fear of COVID-19 scale |  | |  |  |  |
| Is the mother afraid of COVID-19? | Yes | | 165 (42.7) | 285 (38.1) | 0.027^*^ |
|  | No | | 221 (57.3) | 463 (61.9) |  |
| Does it make the mother uncomfortable to think of COVID-19? | Yes | | 165 (42.7) | 243 (32.5) | <0.001^*^ |
|  | No | | 221 (57.3) | 505 (67.5) |  |
| Do you worry a lot about COVID-19? | Yes | | 141 (36.5) | 156 (20.9) | <0.001^*^ |
|  | No | | 245 (63.5) | 592 (79.1) |  |
| Does your hand become clammy when you thin about COVID? | Yes | | 126 (32.6) | 188 (25.1) | 0.002^*^ |
|  | No | | 260 (67.4) | 560 (74.9) |  |
| When watching social media about COVID, does it make you nervous? | Yes | | 110 (28.5) | 148 (19.8) | <0.001^*^ |
|  | No | | 276 (71.5) | 600 (80.2) |  |
| Are you deprived from sleep because of COVID-19? | Yes | | 59 (15.3) | 83 (11.1) | 0.043^*^ |
|  | No | | 327 (84.7) | 665 (88.9) |  |
| Does COVID give you palpitation? | Yes | | 69 (17.9) | 67 (9.0) | <0.001^*^ |
|  | No | | 317 (82.1) | 681 (91.0) |  |
| Total fear of COVID score | | Mean±SD | 9.16±2.393 | 8.56±2.077 | <0.001^*^ |
